# Effectiveness of psychotherapeutic, pharmacological, and combined treatments for chronic depression: a systematic review (METACHRON)

**DOI:** 10.1186/1471-244X-10-95

**Published:** 2010-11-23

**Authors:** Levente Kriston, Alessa von Wolff, Lars Hölzel

**Affiliations:** 1 Department of Medical Psychology, University Medical Center Hamburg-Eppendorf, Germany; 2 Department of Psychiatry and Psychotherapy, University Medical Center Freiburg, Germany

## Abstract

**Background:**

Chronic depressions represent a substantial part of depressive disorders and are associated with severe consequences. Several studies were performed addressing the effectiveness of psychotherapeutic, pharmacological, and combined treatments for chronic depressions. Yet, a systematic review comparing the effectiveness of multiple treatment options and considering all subtypes of chronic depressions is still missing.

**Methods/Design:**

Aim of this project is to summarize empirical evidence on efficacy and effectiveness of treatments for chronic depression by means of a systematic review. The primary objectives of the study are to examine, which interventions are effective; to examine, if any differences in effectiveness between active treatment options exist; and to find possible treatment effect modifiers. Psychotherapeutic, pharmacological, and combined treatments will be considered as experimental interventions and no treatment, wait-list, psychological/pharmacological placebo, treatment as usual, and other active treatments will be seen as comparators. The population of patients will include adults with chronic major depression, dysthymia, double depression, or recurrent depression without complete remission between episodes. Outcomes of the analyses are depressive symptoms, associated consequences, adverse events, and study discontinuation. Only randomized controlled trials will be considered.

**Discussion:**

Given the high prevalence and serious consequences of chronic depression and a considerable amount of existing primary studies addressing the effectiveness of different treatments the present systematic review may be of high relevance. Special attention will be given to the use of current methodological standards. Findings are likely to provide crucial information that may help clinicians to choose the appropriate treatment for chronically depressed patients.

## Background

Chronic depressions represent a substantial part of depressive disorders. Usually four subtypes of chronic depression are distinguished: (1) dysthymia, (2) chronic major depression, (3) recurrent major depression with incomplete remission during episodes, and (4) double depression [1]. Dysthymic disorder is defined as a mild condition that is chronic and persistent for at least 2 years. Major depressive episode, chronic type, refers to a more severe condition that meets full criteria for major depression continuously for a minimum of 2 years. Patients who have recovered to the point where they no longer meet full criteria for a major depressive episode but continue to experience significant symptoms for a total duration of illness greater than 2 years are referred to as recurrent major depression with incomplete remission during episodes. The superimposition of a major depressive episode on antecedent dysthymia is referred to as double depression [1].

There is evidence from diverse studies that about 20% of all patients diagnosed with major depression develop a chronic course [2,3]. Even after five years 12% of the patients still remain depressive [4]. The mean length of chronic depression is approximately 17 to 30 years [3,5]. The lifetime prevalence rate for dysthymia in the US is estimated to be 6% and the one-year prevalence rate around 3% [6]. Among psychiatric outpatients up to 36% suffer from dysthymia [7].

Chronic depression is in comparison with acute depression associated with longer treatment duration, increased loss of physical wellbeing, increased co-morbidity, more severe impairments in social, psychological, and emotional functioning, increased health care utilisation, and more frequent suicide attempts and hospitalisations [1,8].

Several studies were performed addressing the effectiveness of psychotherapeutic and pharmacological treatments of chronic depressions [9-16]. Often the effectiveness of combinations of pharmacological and psychotherapeutic treatments was assessed with promising results [14,17-19].

The effectiveness of psychotherapy for the treatment of chronic depression is only summarised by one older review. The review is mainly based on uncontrolled and non-randomized studies of cognitive behavioral treatments and focuses solely on dysthymia [20]. Because psychotherapeutic treatments for chronic depression have been the subject of several recent studies an up-to-date review is urgently required. For some subtypes of chronic depression the effectiveness of pharmacological treatments has already been subject of systematic reviews [21,22]. A high-quality narrative review also considered combination treatments [23].

A systematic review considering the whole spectrum (i.e. all subtypes) of chronic depressions and a wide variety of available treatment options is still missing.

### Objectives

We aim to summarize empirical evidence on efficacy and effectiveness of treatments for chronic depression by means of a systematic review. The primary objectives of the study are 1) to examine, which interventions are effective; 2) to examine, if any differences in effectiveness of active treatments exist; and 3) to find possible treatment effect modifiers.

## Methods/Design

### Criteria for selecting studies for this review

Following a screening of the titles and abstracts, the decision about inclusion/exclusion will be based on the review of full texts and made independently by two reviewers. If disagreement occurs, it will be recorded and resolved by discussion. Agreement between reviewers will be quantified and reported.

#### Types of studies

To ensure a high internal validity of the findings only randomized controlled trials (RCTs) will be included in the systematic review. The description of the studies as "randomized" will be sufficient for inclusion; however, the method of randomization will be addressed in detail during the assessment of methodological quality. No restrictions regarding time of follow-up or other design characteristics will be applied.

#### Types of participants

Studies conducted in adults with a diagnosis of chronic depression will be included. Trials investigating patients with chronic major depression, dysthymia, double depression, or recurrent depression without a complete remission between episodes will be included if the target disorders are of at least two years' duration. The diagnosis of depression needs to rely on a formal classification system, such as the International Classification of Diseases (ICD) [24] or the Diagnostic and Statistical Manual of Mental Disorders (DSM) [25]. Studies focusing exclusively on chronically depressed patients with concurrent personality disorder, substance abuse, dementia, psychotic disorder, anxiety disorder, or a somatic disorder will be excluded. Studies in which both patients with chronic and acute forms of depression are included will only be considered in the review if data are reported separately for the chronic subgroup.

#### Types of interventions

Psychotherapeutic, pharmacological, and combined interventions will be considered. The interventions have to focus primarily on the treatment of depressive symptoms and need to be acute treatments (maintenance or continuation treatments will be excluded).

Psychotherapeutic interventions have to fulfil the following criteria: 1) the intervention must be based on a scientific theory (in detail described and/or manualized and/or referenced; 2) a minimum of one contact (e.g. session) between therapist and patient must take place (thus, for example the general dissemination of information material in form of leaflets in waiting rooms will not be considered as a psychotherapeutic intervention); and 3) the intervention must consider the personal needs of the patient or a group of patients and must be individually tailored in an interpersonal process (thus, group therapies will be included).

Pharmacological interventions include the administration of pharmacological agents.

Combined interventions include the administration of one or more pharmacological agents combined with one or more psychotherapeutic interventions. Special attention will be paid to the clear description of the so called "medical management" (i.e. the monitoring of a pharmacotherapy), which in some cases may approach the intensity of supportive psychotherapy and can be considered as a stand-alone psychotherapeutic intervention.

Somatic (e.g. electroconvulsive therapy, vagus nerve stimulation, acupuncture), non-pharmacological (e.g. physical exercise, bright light therapy), and organizational (e.g. case management) interventions will not be considered.

#### Types of comparator(s)

Both controlled and comparative effectiveness studies will be included. The comparators may be 1) no-treatment control (patients are administered only assessments); 2) wait-list control (patients receive the treatment following the study period); 3) attention-placebo, nonspecific control, sham treatment (patients receive a treatment that involves nonspecific psychotherapeutic factors); 4) pharmacological placebo (patients receive placebo pills); 5) treatment us usual; 6) other psychotherapeutic treatment; 7) other pharmacological treatment; or 8) other combined psychotherapeutic/pharmacological treatment.

Comparisons with no-treatment and wait-list controls as well as psychotherapeutic or pharmacological placebos may provide information on the efficacy of the investigated interventions, while studies comparing two active treatments possibly allow a ranking of the interventions according to their effectiveness. Trials comparing a pharmacological or psychotherapeutic treatment with a combined intervention may inform about the additional (incremental) effects of certain treatment components.

#### Types of outcome measures

The *primary efficacy outcome *will be response to treatment. It is usually defined as an at least 50% decrease of a depression scale (such as the Hamilton Depression Rating Scale [HDRS] [26] or the Montgomery -Åsberg Depression Rating Scale [MADRS] [27]) score from baseline to end of treatment or achieving a status of "much improved" or "very much improved" on the Clinical Global Impression [CGI] [28] instrument after treatment. If studies report more than one of these measures, priority will be given to HDRS over MADRS and MADRS over CGI. If none of these measures were used, other psychometrically sound observer-rated depression scales may be used to define response. If no observer-rated scale was used, response definitions using self-rated scales (e.g. Beck Depression Inventory [BDI] [29]) may be included.

*Secondary efficacy outcomes *will include metric outcomes of depression scales (e.g. scores on HDRS or BDI), metric outcomes on global scales (e.g. Symptom Checklist-90 [SCL-90] [30]), and dichotomous outcomes other than response, such as remission (e.g. reporting a severity score on a depression scale below a threshold of clinical significance at the end of treatment), relapse (e.g. exceeding a threshold on a depression scale in the follow-up period after having reached a remission after treatment), sustained response (e.g. response without relapse in the follow-up period), or reporting preference for continuation of the received treatment. Further efficacy outcomes may include assessment of impairment and consequences, such as satisfaction with treatment (e.g. Patient Satisfaction Questionnaire [PSQ] [31]), general assessment of functioning (e.g. General Assessment of Functioning [GAF] [25]), or quality of life (e.g. WHO Quality of Life [WHOQOL] [32]).

Efficacy outcomes will be analyzed separately for short- (up to 3 months), medium- (3 to 12 months), and long- (at least 12 months) term.

The *primary safety outcome *will be dropping out of the study due to any reason.

*Secondary safety outcomes *will include treatment-related drop-out from the study (e.g. due to inefficacy of treatment or due to side-effects, respectively), experiencing any adverse event, experiencing any side-effect, and suicidal report/behaviour.

All outcomes that are likely to be meaningful to people making a decision about the target condition (clinicians, patients/consumers, the general public, administrators and policy makers) will be addressed independently of the frequency of their reporting in primary studies [33]. Due to the long tradition of depression research most instruments used in clinical trials are usually psychometrically sound. Such measures will be preferred throughout the review (either referenced and/or sufficient psychometric quality reported).

### Search methods for identification of studies

Several methods will be used to retrieve potentially relevant articles. In addition to standard electronic medical databases also clinical trial registers will be searched. Furthermore, handsearch in relevant journals will be performed. The ancestry approach will be applied through examining reference lists and performing citation searches. In addition, experts will be contacted.

#### Bibliographic database search

The following databases will be searched: Cochrane Central Register of Controlled Trials (CENTRAL), MEDLINE, EMBASE, ISI Web of Science, BIOSIS, PsycINFO, and CINAHL. No language restrictions will be applied. All databases will be searched from 1970 using both standard vocabulary (e.g. Medical Subject Headings [MeSH]) and keywords (freetext). For searches a disease-component will be combined (AND) with a design-component. The population of interest (disease-component) will be identified by combining (AND) terms referring to depression (e.g. depress$ [keyword] OR mood disorders [MesH] OR dysthymi$ [keyword]) with terms referring to chronic states (e.g. chroni$ [keyword]). RCTs (design-component) will be identified using the Cochrane Highly Sensitive Search Strategy for identifying randomized controlled trials [33].

#### Search in clinical trial registers

Clinicaltrials.gov, the International Clinical Trials Registry Platform (ICTRP), and the German Clinical Trial Register (Deutsche Register Klinischer Studien [DRKS]) will be searched for ongoing or non-published studies.

#### Handsearch

A series of journals will be handsearched beginning with the year 1970. The selection was based on scientific impact (impact factor) and focus of the journals: Archives of General Psychiatry, Journal of Consulting and Clinical Psychology, and Journal of Affective Disorders.

#### Ancestry approach

Reference lists of all included studies will be searched. Cited reference search in Social Sciences and Science Citation Index will be performed for all included studies.

#### Expert contacts

The first author of all included studies will be contacted for further information regarding published and unpublished trials.

### Data collection and assessment of methodological quality

#### Data extraction

Study characteristics and results will be extracted independently by two reviewers using a structured form. Disagreement will be resolved by discussion.

Outcomes will be extracted from publications with estimation and substitution of missing data according to the guidelines of the Cochrane Collaboration [33], e.g. calculating standard errors from exactly reported t-values. The primary efficacy outcome (response) will be estimated from appropriate metric variables if it is not reported in the study [34].

#### Assessment of methodological quality

The Cochrane Collaboration's tool for assessing risk of bias will be used to assess *internal validity *of the included studies [33]. This tool addresses sequence generation; allocation concealment; blinding of participants, personnel, and outcome assessors; incomplete outcome data; selective outcome reporting; and other sources of bias. An overall assessment of bias will be performed by classifying all studies in the categories of low, unclear, and high risk of bias.

*External validity *(generalizability) will be addressed by documenting study setting, patient selection criteria, patient characteristics, applicability of the intervention in routine care, clinical relevance of outcomes, length of follow-up, adverse effects, and discontinuation rates.

Study quality will be assessed independently by two reviewers. Disagreement will be recorded and resolved by discussion. If considerable methodological heterogeneity is present, subgroup analyses will be performed by comparing the findings between studies of low, unclear, and high risk of bias.

### Data synthesis

#### Planned treatment comparisons

Psychotherapeutic treatments will be grouped into 1) behavioural and cognitive behavioural therapies; 2) humanistic therapies; 3) interpersonal, cognitive analytic, and other integrative therapies; 4) mindfulness-based, 'third wave' therapies; and 5) psychodynamic therapies. Pharmacological treatments will be grouped into 1) tricyclic antidepressants; 2) monoamine oxidase inhibitors; 3) selective serotonin reuptake inhibitors; 4) 'third-generation' antidepressants; and 5) other pharmacological treatments. Combination treatments will be considered as the combination of any psychotherapeutic with any pharmacological treatment class. For primary analyses, comparators will be grouped into control and active interventions. Although the combination of these classes implies a high number of theoretically possible comparisons, qualitative evidence suggests that only an extremely limited number of them has been performed [23], thus keeping the number of comparisons manageable.

Secondary analyses will address both more global (e.g. psychotherapy vs. pharmacotherapy) and more specific (e.g. different agents within the class of selective serotonin reuptake inhibitors) comparisons.

#### Meta-analysis

The statistical analysis will follow actual guidelines [33,35,36]. Effectiveness measures for dichotomous outcomes will be benefit and risk ratios (depending on the beneficial/adverse character of the outcome). For rare outcomes (adverse events, and possibly drop-out rates) or endpoints with highly varying baseline rates odds ratios will be calculated. For commonly used instruments, such as the HDRS and the BDI, mean difference (previously called weighted mean difference) will be calculated, which assumes the utilization of the same scale across studies. For other metric measures (e.g. quality of life) standardized mean difference will be calculated, as it is unlikely that all studies administer the same measures. For all studies, effect sizes will be calculated using the intention-to-treat principle, i.e. analyzing all subjects allocated to a study arm. For the primary outcomes (both efficacy and safety) all randomized patients will be included in the analyses irrespective of how the authors of the primary studies defined their intention-to-treat sample. For the primary efficacy outcome (response to treatment) it means that all discontinuations from the point of randomisation will be considered as non-response. For all other (mostly metric) outcomes the definition of the intention-to-treat sample provided by the authors will be followed. The most common approach in primary studies is probably a 'modified' intention-to-treat analysis excluding only patients that dropped out before the first or second visit and imputing outcome data using the 'last observation carried forward' (LOCF) principle for all other discontinuations.

All analyses will be preformed by applying a random effects model with inverse variance weights [37]. We plan to use a random effects model rather than fixed effects one, because we assume that the included studies will not be functionally equivalent and will show considerable clinical (concerning population, intervention) and methodological (design, quality etc.) heterogeneity. Statistical heterogeneity between study results will be tested for significance using Cochran's Q-test and quantified using the I^2 ^statistic [38]. Results will be visually displayed as forest plots.

Possible publication bias will be tested using visual examination of funnel plots and applying Egger's test [39].

#### Subgroup and meta-regression analysis

A priori defined subgroup (in case of categorical predictors) or meta-regression (in case of metric predictors) analyses will be performed according to the subtype of chronic depression, duration (onset) and severity of the target disorder, as well as study quality. Differences between subgroups will be tested formally [40-42]. All meta-regression analyses will be performed using the restricted maximum likelihood estimate method, a recommended random effect approach that accounts for residual between-trial heterogeneity [43].

In case of considerable heterogeneity between study results that cannot be explained by the a priori defined subgroup and meta-regression analyses, a series of a posteriori (explorative) meta-regression analyses will be performed to identify sources of heterogeneity. A priori and a posteriori analyses will be clearly labelled as such.

#### Sensitivity analysis

Sensitivity analyses will be performed for the primary efficacy outcome using per-protocol data. Results will be contrasted to those acquired pooling intention-to-treat effect sizes.

#### Network meta-analysis

Due to recent developments in meta-analytical methods a new procedure has become available to synthesize evidence from direct and indirect comparisons of interventions, which allows the assessment of the relative effectiveness of two treatments even if they have not been compared directly in a randomized trial [44,45]. These so called network or mixed treatment meta-analyses estimate the relative effectiveness of two treatments from all available direct and indirect evidence that is present in a network of treatments and comparisons, and thus suggest a ranking of interventions according to their relative effectiveness. We aim to perform a network meta-analysis to create a hierarchy of all available treatments for chronic depression, provided the collected data allow for it.

#### Qualitative summary

If clinical and/or methodological heterogeneity of the included studies proves to be extremely high, a qualitative rather than quantitative synthesis of the evidence will be performed [33,36].

## Discussion

With generating the present review we provide a complete report and meta-analytical comparison of the evidence for psychotherapeutic, pharmacological, and combined treatments for chronic depression. Additional subgroup analyses and meta-analytical exploration of treatment effect modifiers shall reveal whether different subtypes of chronic depression should be treated in different ways. This information may help the clinicians to choose the right treatment for chronically depressed patients. Given the high prevalence and serious consequences of chronic depression as well as a considerable amount of existing primary studies addressing effectiveness of different treatments, the present systematic review may be of high relevance. Special attention will be given to the use of current methodological standards.

Findings of the review will be disseminated as publications in scientific journals. The articles will be prepared according to the Preferred Reporting Items for Systematic Reviews and Meta-analyses (PRISMA) statement [46]. Summary primary outcome data for each study will be published in order to allow for a re-analysis by independent research groups. If changes or amendments to this study protocol are made, they (including rationale) will be reported. A bilingual (English and German) study website will be implemented to disseminate further details and materials.

## Competing interests

The authors declare that they have no competing interests.

## Authors' contributions

LK formulated the research question, sketched the research design, defined the statistical methods, and drafted the manuscript. AW participated in the development of the research design, reviewed existing literature, and revised the manuscript substantially. LH participated in formulating the research question, in the design and coordination of the study, as well as in drafting the manuscript. All authors read and approved the final manuscript.

## Pre-publication history

The pre-publication history for this paper can be accessed here:

http://www.biomedcentral.com/1471-244X/10/95/prepub
